# Elective nodal radiotherapy with a gapless radiation field junction for oligorecurrent prostate cancer after previous radiotherapy

**DOI:** 10.1016/j.ctro.2022.100571

**Published:** 2022-12-24

**Authors:** Minglun Li, Yourong Fan, Christian Trapp, Nina Sophie Schmidt-Hegemann, Jing Ma, Alexander Buchner, Shun Lu, Bin Xu, Christian Stief, Xuanbin Wang, Cheng Zhou, Claus Belka, Paul Rogowski

**Affiliations:** Ludwig Maximilians University LMU University Hospital Munich, Germany

**Keywords:** ADT, androgen deprivation therapy, CTCAE, common terminology criteria for adverse events, CTV, clinical target volume, D_1cc-cum_, maximum cumulative dose in 1 cc, D_max-cum_, cumulative maximum dose, ENRT, elective nodal radiotherapy, EQD_2_, equivalent dose in 2 Gy fractions, IGRT, image-guided radiotherapy, IMRT, intensity-modulated radiotherapy, LN, lymph nodes, OAR, organs at risk, PSA, prostate-specific antigen, PSMA-PET/CT, prostate-specific membrane antigen positron emission tomography/computed tomography, RT, radiotherapy, SBRT, stereotactic body radiotherapy, SIB, simultaneous integrated boost, VMAT, volumetric modulated arc therapy, ENRT, Nodal oligorecurrence, Gapless radiation field junction, Reirradiation, PSMA-PET/CT

## Abstract

**Purpose:**

To evaluate the feasibility of subsequent elective nodal radiotherapy (ENRT) for nodal recurrences after previous radiotherapy with a defined planning approach for a gapless radiation field junction.

**Methods:**

Patients with 1) previous radiotherapy of prostate or prostatic fossa and subsequent pelvic ENRT or 2) previous pelvic radiotherapy and subsequent ENRT to paraaortic lymph nodes (LN) and gapless junction of both radiation fields were analyzed. The cumulative maximum dose (D_max-cum_) and the maximum cumulative dose in 1 cc (D_1cc-cum_) were estimated. Absolute toxicity and the toxicity exceeding baseline were evaluated.

**Results:**

Twenty-two patients with PSMA-PET/CT-staged nodal oligorecurrence after prior radiotherapy were treated with pelvic (14 patients) or paraaortic ENRT (9 patients). One patient was treated sequentially at both locations. Median time between first and second RT was 20.2 months. Median doses to the lymphatic pathways and to PET-positive LN were 47.5 Gy and 64.8 Gy, respectively. The planning constraint of an estimated D_max-cum_ ≤ 95 Gy and of D_1cc-cum_ < 90 Gy were achieved in 23/23 cases and 22/23 cases, respectively. Median follow-up was 33.5 months. There was no additional acute or late toxicity ≥ grade 3. Worst acute toxicity exceeding baseline was grade 1 in 68.2% and grade 2 in 22.7% of patients. Worst late toxicity exceeding baseline was grade 1 in 31.8% and grade 2 in 18.2% of patients.

**Conclusion:**

ENRT for nodal recurrences after a previous radiotherapy with gapless junction of radiation fields seems to be feasible, applying the dose constraints D_max-cum_ ≤ 95 Gy and D_1cc-cum_ < 90 Gy without grade 3 acute or late toxicities exceeding baseline.

## Highlights

• A gapless junction of an elective nodal radiotherapy field after previous radiotherapy is feasible.
• A cumulative maximum dose (D_max-cum_) ≤ 95 Gy and a maximum cumulative dose in 1 cc (D_1cc-cum_) < 90 Gy were used as dose constraints.
• This approach is safe without additional acute or late grade 3 toxicity.

## Background

Pelvic and/or paraaortic oligometastatic lymph node (LN) recurrence is a frequent pattern of relapse after primary treatment in men with prostate cancer [1], [2], [3]. Indeed, with prostate-specific membrane antigen positron-emission tomography/computed tomography (PSMA PET/CT) currently suggested by several international guidelines for staging after biochemical relapse, the rate of diagnosed oligometastatic LN recurrences is expected to increase [4]. Radiotherapy (RT) offers an effective and safe therapeutic option in this situation, besides salvage pelvic lymph node dissection and palliative systemic treatments [5], [6], [7].

However, if patients had previous RT to the prostate, the prostatic fossa and/or the pelvis during their disease course, the recurrent LN metastases can be in close proximity to the prior irradiation treatment field. Hence, there are concerns that a repeated RT with adjacent or overlapping treatment fields leads to excess toxicity, because small bowel, rectum, sigmoid and bladder are at risk of being partially re-irradiated. Thus, in order to minimize the volume of overlapping doses a focal lesion-directed radiotherapeutic approach using stereotactic body radiotherapy (SBRT) is frequently chosen in this setting over a more comprehensive approach including uninvolved LN regions using elective nodal radiotherapy (ENRT) [8], [9]. Nevertheless, a lesion-directed approach does not cover microscopic disease in the vicinity of the macroscopic LNs and retrospective data suggests, that locoregional control is improved with ENRT [10], [11], [12].

Literature describing ENRT for pelvic or paraaortic nodal recurrences after prior adjacent RT is sparse. In Oligopelvis GETUG P07, a phase 2 trial investigating salvage RT and hormone therapy in pelvic nodal oligorecurrent prostate cancer, previous irradiation of the prostate or the prostatic fossa was allowed, provided that there was a minimum of 1 cm gap between the prostate and salvage pelvic radiotherapy fields [13], [14]. No increased early or late toxicity in patients with previous radiotherapy to the prostatic fossa was observed. However, details on the planning techniques and risk assessment for organs at risk have not been published yet.

Aims of this study were 1) to evaluate the feasibility of a subsequent ENRT for nodal recurrences after a previous adjacent RT with a defined planning approach for a gapless radiation field junction; 2) to report the estimated cumulative doses to the organs at risk (OAR) and the toxicity in these patients.

## Methods

### Patient population

Consecutive patients undergoing salvage radiotherapy for pelvic or paraaortic nodal recurrent prostate cancer at the University hospital, LMU Munich, were retrospectively identified. Patients with 1) a previous irradiation of the prostate or the prostatic fossa and a subsequent pelvic ENRT or 2) a previous pelvic RT and a subsequent ENRT to paraaortic LN region and potential overlapping treatment fields were considered for analysis. Patients without follow-up information were excluded. All patients had histologically confirmed prostate cancer and were referred due to a rising prostate specific antigen (PSA). Treatment indications were approved by an interdisciplinary tumor board. This retrospective analysis was performed in compliance with the principles of the Declaration of Helsinki and its subsequent amendments [15] and was approved by the local Ethics Committee of the Medical Faculty (approval number 19–361).

### Cumulative dose estimation and treatment planning

**Fig. 1:**
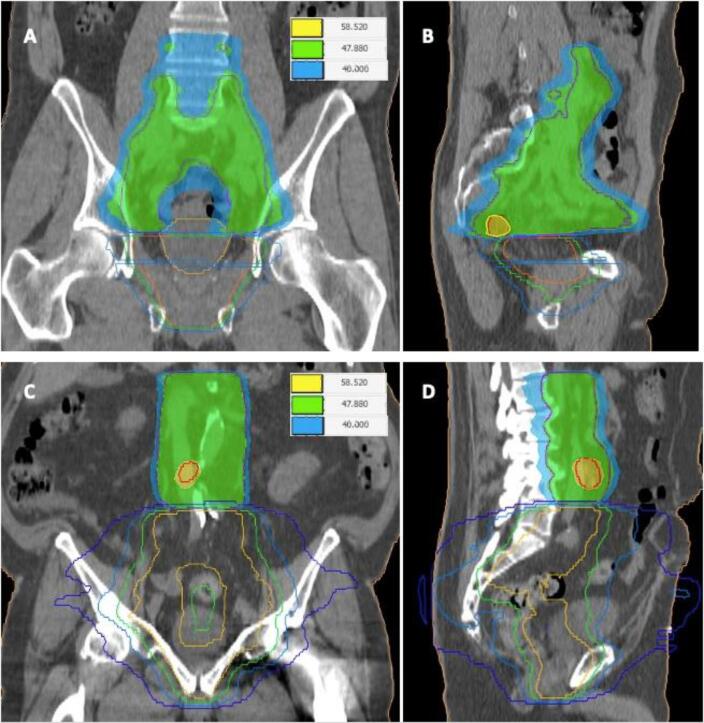
Examples of a patient treated with pelvic enrt after previous rt of the prostatic fossa (a + B) and a patient treated with paraaortic ENRT after previous RT of the pelvis (C + D). Isodose contours of the previous RT: dark blue = 20 Gy, light blue = 30 Gy, green = 40 Gy, orange 50 Gy. A + C: coronar views, B + D sagittal views. (For interpretation of the references to colour in this figure legend, the reader is referred to the web version of this article.)

First, representative isodose lines from the previous treatment (typically, isodoses for 20 Gy, 30 Gy, 40 Gy and 50 Gy) were transferred into the actual planning-CT as contours (see also Fig. 1). Second, the maximum dose (D_max_) and the maximum dose in 1 cc (D_1cc_) from the second RT within each isodose were calculated. Third, the value of the respective isodose was summed with D_max_ or the D_1cc_, respectively, to calculate the cumulative maximum dose (D_max-cum_) and the maximum cumulative dose in 1 cc (D_1cc-cum_) (see also Fig. 1). A D_max-cum_ ≤ 90 Gy and a D_1cc-cum_ ≤ 80 Gy were defined as planning aims, a D_max-cum_ ≤ 95 Gy and a D_1cc-cum_ ≤ 90 Gy were defined as hard constraints.

### Radiotherapy treatment and follow-up

All patients were treated with intensity-modulated radiotherapy (IMRT) or volumetric modulated arc therapy (VMAT) with 5 fractions per week. Image-guided radiotherapy (IGRT) was performed with cone-beam CT daily. RT dose regimens were normo- or slightly hypofractionated with a simultaneous integrated boost (SIB) to the pathological LNs. Target delineation for pelvic lymphatic drainage pathways was performed according to NRG Oncology Updated International Consensus Atlas on Pelvic Lymph Node Volumes [16]. Clinical target volume (CTV) for paraaortic ENRT encompassed a 7 mm margin around the great blood vessels carving out bone, muscle and OAR. Cranially, the border was chosen 2–4 cm above the highest pathological LN. The same procedure was chosen caudally. However, care was taken that the CTV did not directly overlap with the previous PTV. ADT was recommended to all patients for 24–36 months, but the duration could be adjusted at the discretion of the treating urologist depending on comorbidities, side effects, and patient’s preference. Follow-up examination was first carried out 3 months after RT and then every-six to 12 months. Baseline (before second RT), the worst acute (occurring during the second RT course and in the following three months) and worst late (occurring thereafter) toxicities were reported based on the Common Terminology Criteria for Adverse Events (CTCAE) Version 5.0. Furthermore, because baseline toxicity was high in this patient collective with multimodal pretreatments, acute and late toxicity exceeding baseline were also assessed.

### Statistical analysis

Descriptive statistics were used to describe patient and treatment characteristics. Continuous measures were summarized using median and range, whereas ordinal and categorial measures were summarized using counts and percentages. Baseline and acute/late toxicity were compared using the Wilcoxon rank sum test for matched pairs. Differences in the toxicity profile between the group treated with pelvic ENRT and the group treated with paraaortic ENRT were tested using the Mann-Whitney-U-Test. Correlation between dosimetric data and toxicity was assessed using the Spearman rank test. P-values of < 0.05 were considered as statistically significant.

## Results

One hundred eighteen consecutive patients undergoing salvage RT for pelvic or paraaortic LN recurrence between January 2014 and December 2020 were screened for previous RT with adjacent treatment fields. In total, 23 patients fulfilled the inclusion criteria. One patient was excluded due to missing follow-up information. Hence, 22 patients were eligible for analysis.

Patient and treatment characteristics are shown in Table 1. The median age at the time of the second RT was 73.5 years (range 57–81). Fourteen patients had prior adjuvant or salvage RT of the prostate fossa (n = 13, 59.1%) or definitive RT of the prostate (n = 1, 4.5%) and were treated with subsequent ENRT of the pelvic lymphatic drainage pathways due to pelvic nodal recurrence. Nine patients (40.9%) had prior pelvic RT (with or without a boost on pathological LN) with a boost on the prostatic fossa and were treated with ENRT of the paraaortic lymphatic drainage pathways. One patient treated first with pelvic ENRT in our institution after previous salvage RT to the prostate fossa had another nodal recurrence in the area of the aortic bifurcation and received an additional ENRT of the paraaortic lymphatic drainage pathways, which explains the discrepancy between patient number (n = 22) and sum of treatments (n = 23). Median time between the first and the second RT was 20.2 months (range 7.0–136.2 months). Staging prior to the second RT was conducted with PSMA-PET/CT in all patients. The number of PET-positive LN-metastases was one in 60.8% and two in 39.2% of RT series. Median RT doses converted to EQD_2_ equivalent doses using an α/β ratio of 1.5 Gy were 47.5 Gy (range 42.4–47.5 Gy) to the lymphatic drainage pathways with a SIB to PET-positive LN of 64.8 Gy (range 56.0–65.1 Gy). ADT was recommended to all patients but refused by six patients, resulting in concomitant administration in 73.9% of RT series.

**Table 1:** Patient characteristics.

Patients, n	22
Age (years), median (range)	73.5 (57–81)
Initial tumor stage, n (%)	
T2	5 (22.7)
T3	17 (77.3)
Initial nodal stage n (%)	
N0	18 (81.8)
N1	3 (13.6)
Nx	1 (4.5)
Initial ISUP score, n (%)	
2	2 (9.1)
3	9 (40.9)
4	6 (27.3)
5	5 (22.7)
Initial PSA (ng/ml), median (range)	13.5 (5–91)
RT1, n (%)	
Definitive RT of prostate	1 (4.5)
Postoperative RT of prostatic fossa	13 (59.1)
Postoperative pelvic RT + boost of prostatic fossa	9 (40.9)
Time between RT1 and RT2 (months), median (range)	20.2 (7.0–136.2)
Imaging before RT2	
PSMA-PET/CT	23 (100)
RT2, n (%)	
Pelvic RT	14 (63.6)
Paraaortic RT	9 (40.9)
Number of LN in PSMA-PET/CT (n), median (range)	1 (1–2)
ADT during RT2, n (%)	
yes	17 (73.9)
no	6 (26.1)
Abbreviations: ADT = androgen deprivation therapy; ISUP = International Society of Urological Pathology; LN = lymph node; PSA = prostate-specific antigen; PSMA-PET/CT = prostate-specific membrane antigen positron emission tomography/computed tomography; RT = radiotherapy	

Table 2 shows the estimation of the cumulative doses. The planning goal of an estimated D_max-cum_ ≤ 90 Gy was achieved in 18/23 cases (78.2%). The planning goal of D_1cc-cum_ < 80 Gy was achieved in 13/23 cases (56.5%). The hard constraints of D_max-cum_ ≤ 95 Gy and D_1cc-cum_ < 90 Gy were achieved in 22/23 (95.6%) and 23/23 (100%) cases, respectively.

**Table 2:** Estimation of cumulative maximum doses.

	D_max-cum_, median (range)	D_1cc-cum_, median (range)
Volume iso20	70.8 Gy (46.0-84.8)	68.0 Gy (43.6-78.3)
Volume iso30	77.4 Gy (41.6-90.3)	70.0 Gy (40.9-83.5)
Volume iso40	78.3 Gy (42.2-93.9)	70.9 Gy (42.2-92.4)
Volume iso50	80.0 Gy (52.1 - 110.9)	75.0 Gy (50.4-89.8)

Volume iso20 = the volume encompassing the 20 Gy isodose of the previous RT. In some cases, nearby isodoses were chosen, if the information on the exact 20/30/40/50 Gy isodose was not available.

**Fig. 2:**
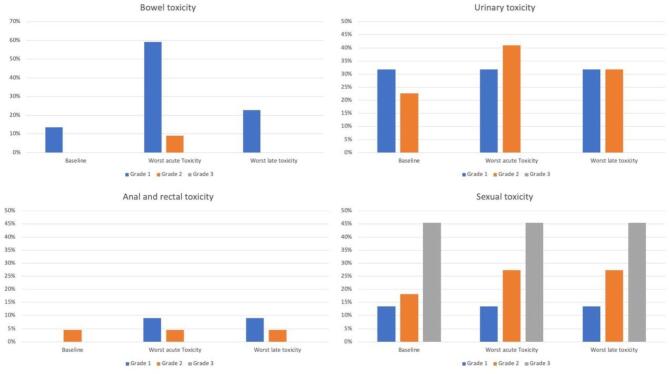
Baseline, acute and late toxicity.

All patients completed the salvage RT as planned. Median follow-up was 33.5 months. Treatment related toxicity is summarized in Table 3, Table 4 and Fig. 2. There was no additional acute or late toxicity grade ≥ 3 observed. The most frequent acute toxicity was diarrhea (59.1% grade 1, 9.1% grade 2), which resolved in most cases within 3 months post RT. There was a significant difference between baseline bowel symptoms and acute toxicity (p < 0.001), but not between baseline and late toxicity (p = 0.157). The acute rectal toxicity consisted of mild proctitis (9.1% grade 1, 4.5% grade 2). No late bowel toxicity grade ≥ 2 exceeding baseline was observed. Acute urinary toxicity consisted mainly of urinary incontinence, frequency and urgency (27.3% grade 1, 45.4% grade 2), which persisted as late toxicity grade 1 in 31.8% and grade 2 in 36.4% of patients. There was a significant difference in acute or late urinary toxicity, compared to baseline, respectively. However, baseline urinary symptoms were frequent and after adjusting for toxicity exceeding baseline, acute urinary toxicity were grade 1 in 22.7% and grade 2 in 9.1%, respectively. Late urinary toxicity exceeding baseline was grade 1 in 18.2% and grade 2 in 4.5%, respectively. One patient with a previous pelvic RT plus boost on the prostatic fossa treated with subsequent RT of the paraaortic lymphatic drainage pathways developed a urethral stenosis in the area of the anastomosis during follow-up and, because the treated field was clearly cranially of the urethra, development of the stenosis is rather attributable to prostatectomy and/or the first RT than to the second RT. We observed only a mild increase in erectile dysfunction. One patient suffered from a symptomatic insufficiency fracture of the pubic bone, which was treated with analgesics. Any toxicity taken together, the acute toxicity exceeding baseline was grade 1 in 68.2% and grade 2 in 22.7% of patients and the late toxicity exceeding baseline was grade 1 in 31.8% and grade 2 in 18.2% of patients.

**Table 3:** Absolute acute and late toxicity.

	Baseline		Acute toxicity		Late toxicity		p
	n	%	n	%	n	%	
Bowel toxicity							p <.001 acute toxicity vs baseline, p =.157 late toxicity vs baseline
Grade 1	3	13.6	13	59.1	5	22.7	
Grade 2	–	–	2	9.1	–	–	
Grade 3	–	–	–	–	–	–	
Rectal toxicity							p =.157 acute toxicity vs baseline, p =.157 late toxicity vs baseline
Grade 1	–	–	2	9.1	2	9.1	
Grade 2	1	4.5	1	4.5	1	4.5	
Grade 3	–	–	–	–	–	–	
Urinary toxicity							p =.033 acute toxicity vs baseline vs, p =.034 late toxicity vs baseline
Grade 1	7	31.8	6	27.3	7	31.8	
Grade 2	5	22.7	10	45.4	8	36.4	
Grade 3	–	–	–	–	–	–	
Sexual toxicity							p =.157 acute toxicity vs baseline, p =.157 late toxicity vs baseline
Grade 1	3	13.6	3	13.6	3	13.6	
Grade 2	4	18.2	6	27.3	6	27.3	
Grade 3	10	45.5	10	45.5	10	45.5	

When symptoms changed during follow-up, the highest grade was considered. Furthermore, only the highest grade within one category was counted.

**Table 4:** Acute and late toxicity exceeding baseline.

	Acute toxicity		Late toxicity	
	n	%	n	%
Bowel toxicity				
Grade 1	12	54.5	2	9.1
Grade 2	1	4.5	–	–
Grade 3	–	–	–	–
Rectal toxicity				
Grade 1	2	9.1	2	9.1
Grade 2	–	–	–	–
Grade 3	–	–	–	–
Urinary toxicity				
Grade 1	5	22.7	4	18.2
Grade 2	2	9.1	1	4.5
Grade 3	–	–	–	–
Sexual toxicity				
Grade 1	–	–	–	–
Grade 2	2	9.1	2	9.1
Grade 3	–	–	–	–
Any toxicity				
Grade 1	15	68.2	7	31.8
Grade 2	5	22.7	4	18.2
Grade 3	–	–	–	–

For each patient the difference between acute/late toxicity and baseline was assessed.

There was no statistically significant difference for any category of toxicities between the group treated with pelvic ENRT and the group treated with paraaortic ENRT. Furthermore, no correlation was found between estimated dose and acute or late toxicity exceeding baseline.

## Discussion

Randomised trials of postoperative salvage radiotherapy to the prostatic fossa report post-therapeutic biochemical failure rates in excess of 12% to 25% at 5 years [17], [18] with pelvic nodal recurrence being the pattern of failure in 25% to 55% [19], [20], [21]. Furthermore, after previous salvage pelvic radiotherapy, ex-field LN recurrence occurs in 19% to 39% [14]. In both scenarios, ENRT is an effective treatment option. However, the detected LN metastases can be in close vicinity to previous irradiation fields and treatment with repeated RT may lead to overlapping treatment fields. On the other side, occult tumour cells in the adjacent lymph drainage pathways, mostly cranial to the previously irradiated area, present another problematic issue, which makes a gap between both radiation fields a potential risk for relapse. Till now, it remains unclear how the radiation fields and dose distribution should be arranged to those of the previous radiotherapy.

Our retrospective analysis suggests that the presented approach for repeated ENRT with a gapless junction of irradiation fields is feasible without excess toxicity. No additional grade 3 acute or late toxicity occurred and changes in toxicity from baseline were low-grade and reversible in most cases in this cohort, taken the multimodal pre-treatments and the pre-existing symptoms into consideration. Our results are comparable to the reported toxicity data from the prospective Oligopelvis GETUG P07, which also investigated pelvic ENRT in 35 patients after prior RT to the prostate or the prostate fossa: Vaugier et al. and Supiot et al. reported diarrhoea as the most prevalent acute toxicity with grade 1 and 2 toxicity in 55% and 12%, respectively, at one months after RT, which also resolved in most cases after two years [13], [14]. The rate of rectal toxicity grade 1 and 2 was 18% and 3% at one months and 14% and 4% after two years, thus slightly higher compared to our results (9.1% grade 1 and 4.5% late grade 2 acute and late toxicity). In contrast, baseline, acute and late low-grade urinary toxicity was more prevalent in our analysis. However, in Oligopelvis two cases of severe grade 3 incontinence were reported in patients pre-treated with prostatectomy and salvage RT to the prostatic fossa. Differences might be explained due to different planning strategies and constraints. However, these details have not yet been published for Oligopelvis.

In the present study, a multiparameter dose prescription for the cumulative D_max-cum_ and D_1cc-cum_ with soft and hard constraints were performed and evaluated. Using different isodose line contours from the first RT, the dosimetric distribution of the second RT could be prescribed and modified to achieve a gapless conjunction, avoiding an underdosed area and thus reducing the risk of relapse on this site. On the other hand, the predefined cumulative dose could exclude unexpected high dose, limiting the risk of side effects. This pragmatic approach can be seen as a middle course between 1) a pure summation of maximum doses from both radiotherapies, which often leads to a dose overestimation, as the maxima of the first and second RT are usually not at the same point, and 2) a voxel-based dose-summation with co-registration of both plans and deformable image registration, which is in principle more precise, but are technically elaborate and time-consuming. Our approach offers a good dose estimation, is easy to use in daily practice and allows for quick planning. Since with our planning approach there was only a narrow area of dose overlap between the first and second RT, we decided to focus on D_max_ and D_1cc_. Larger volume proportions of the second irradiation, e.g. the maximum dose in 50 cc (D_50cc_), were negligible in almost all cases.

So far, evidence for pelvic re-irradiation mainly consists of retrospective data and applied doses to OARs are inconsistent [22]. A study investigating pelvic stereotactic re-irradiation after conventionally radiotherapy in 27 patients showed no ≥ grade 3 toxicity with median EQD_2_-converted cumulative doses for rectum, bowel and bladder of 104 Gy, 98 Gy and 113 Gy, respectively [23]. In accordance with this report, various studies investigating re-irradiation for rectal cancer have proposed cumulative tolerance doses of 70 Gy to 110 Gy for the rectum [24] and a recent consensus statement for re-irradiation recommends limiting the cumulative D_0.5cc_ to the bladder to 110 Gy [25]. Compared to these dose constraints in literature, our approach limiting the cumulative D_max-cum_ to 90 Gy and the D_1cc-cum_ to 80 Gy seems reasonable, almost conservative, given the fact that OARs in our study were exposed only to a narrow area of dose overlap and no complete reirradiation was involved. However, prospective data on reirradiation in the pelvis and on repair effects to irradiation in pelvic OARs is warranted.

Our approach using ENRT of the pelvic or the *para*-aortic lymphatic drainage pathways, respectively, is an alternative to a focal lesion-directed technique, which is frequently used in the scenario of nodal recurrence after prior irradiation due to a steeper dose gradient towards OARs and has shown a safe toxicity profile in retrospective series [8], [22], [26]. However, recent estimates of human late normal tissue α/β ratios as low as 0.7 and 0.8 for urinary incontinence and haematuria, respectively, indicate that the bladder may be sensitive to high single doses, favouring indirectly a normofractionated approach [27]. Furthermore, with SBRT, PET-negative microscopic disease in the adjacent nodal area is not covered and retrospective data suggests, that locoregional control is improved with ENRT compared to SBRT, albeit at cost of an increase in toxicity [10], [11]. Other possible treatment volumes that range between a strictly focal involved-node SBRT and a ENRT of the entire pelvic or *para*-aortic lymphatic drainage pathways were suggested by some authors (involved site SBRT/involved field RT) [12], [28]. However, these approaches come from single retrospective series and were not compared with other treatment strategies [29], [30]. Therefore, prospective data from trials like PEACE V: STORM comparing SBRT with ENRT [31] are eagerly awaited.

ADT has been proven to be beneficial for nodal metastatic patients and recommended in different guidelines [32], [33]. Based on that, ADT was recommended to all patients in our series, but was refused by six patients, resulting in concomitant administration in 74% of RT series. However, it should be stated that the role of ADT concomitant to RT in the setting of nodal recurrence is still unclear, especially in the case of limited number of lymph nodes, as in the present study with one or two affected lymph nodes. Some retrospective studies reported a positive impact of ADT use concomitant to RT [5], while others showed no benefit [34], [35]. Another trials investigated RT alone with the aim to defer the onset of ADT [36]. For example, Fodor et al demonstrated that patients with nodal oligorecurrence had 25 to 44 months ADT-free survival time after SBRT of lymph nodes. Taken together, more investigations are warranted to set up a more individualized therapy strategy.

Adding prophylactic pelvic radiotherapy as well as extending pelvic radiotherapy fields is under debate for prostate cancer in both definitive and salvage setting: The Phase 3 POP-RT trial showed an improved biochemical failure-free survival and disease-free survival with prophylactic pelvic irradiation in the definitive treatment of high-risk locally advanced prostate cancer [37]. As for biochemical recurrence after prostatectomy, recent results from the NRG Oncology/RTOG 0534 SPPORT trial reported a higher freedom from progression with the addition of pelvic radiotherapy to RT of the prostatic fossa[38]. In addition, pattern of failure analyses after pelvic ENRT have shown that with current treatment field recommendations a relevant proportion of LN are inadequately covered, thus e.g. recommending extending the superior border of the treatment field [39], [40]. However, although prophylactic pelvic RT and extending pelvic treatment fields may benefit some high-risk patients with microscopic tumour cells metastasis in lymph drainage pathways, there might be a proportion of patients who are overtreated, suffering from radiation-induced side effects unnecessarily. Therefore, based on modern imaging with PSMA-PET/CT and the ability of safely adding a pelvic or paraaortic ENRT after prior adjacent RT as shown in our study, a concept of “radiotherapy on demand”, which tailors treatment fields based on imaging findings, might be an option for a more individualized approach. Outcome data of our patient collective will be published separately and will shed further light on this approach.

Nevertheless, as sensitivity of PSMA-PET/CT depends on pre-imaging PSA value and outcome worsens with increasing PSA, sparing treatment until the recurrence becomes visible in PET/CT might be detrimental [41], [42]. Therefore, our results can only be hypothesis-generating and should be investigated prospectively and on a larger scale.

Our study has several limitations, namely its retrospective nature and the small sample size. Additionally, cumulative doses in our approach are an estimation, rather than a precise calculation. However, so far there is only few data on ENRT after prior RT and we believe that our results still deliver some useful evidence for clinical physicians handling similar cases and can serve as basis for further investigations.

## Conclusion

A planning approach with multiparameter dose summation is feasible for elective nodal radiotherapy with gapless conjunction of treatment fields in nodal oligorecurrent prostate cancer after previous radiotherapy. There were no unexpected higher toxicities in the acute or late phase.

## Ethics approval

This retrospective analysis was approved by the local Ethics Committee of the Medical Faculty, LMU Munich, (approval number 19–361).

## Funding

This work was supported by Sino-German Center for Research Promotion project number M-0171.

## Availability of data and materials

Research data are stored in an institutional repository and will be shared upon request to the corresponding author.

## CRediT authorship contribution statement

**Minglun Li:** Conceptualization, Methodology, Supervision, Writing - review & editing. **Yourong Fan:** Data curation. **Christian Trapp:** Methodology. **Nina Sophie Schmidt-Hegemann:** Methodology. **Jing Ma:** Data curation. **Alexander Buchner:** Methodology. **Shun Lu:** Writing – review & editing. **Bin Xu:** Writing – review & editing. **Christian Stief:** Supervision. **Cheng Zhou:** Writing – review & editing. **Claus Belka:** Supervision. **Paul Rogowski:** Conceptualization, Writing – original draft.

## Declaration of Competing Interest

The authors declare that they have no known competing financial interests or personal relationships that could have appeared to influence the work reported in this paper.
